# Sector-specific Long-term Associations Between Transportation, Industrial, and Residential Combustion Air Pollutant Mixtures (PM_2.5_, SO_2_, NO_2_, O_3_) and Neurological Disease-related Mortality in Canada

**DOI:** 10.1097/EE9.0000000000000467

**Published:** 2026-03-19

**Authors:** Kimberly Mitchell, Chris Hebbern, Annalise Ferro, Toyib Olaniyan, Tanya Christidis, Jasmine D. Cakmak, Mathieu Rouleau, Angelos T. Anastasopolos, Ivana Popadic, Markey Johnson, Naizhuo Zhao, Michael Tjepkema, Sabit Cakmak

**Affiliations:** a Environmental Health Science & Research Bureau, Health Canada, Ottawa, Ontario, Canada; b Climate Change and Health Office, Health Canada, Ottawa, Ontario, Canada; c School of Public Health Sciences, University of Waterloo, Waterloo, Ontario, Canada; d Health Analysis and Modelling Division, Statistics Canada, Ottawa, Ontario, Canada; e The Montreal Neurological Institute, McGill University, Montreal, Quebec, Canada; f Water and Air Quality Bureau, Health Canada, Ottawa, Ontario, Canada; g Meteorological Service of Canada, Environment and Climate Change Canada, Dorval, Quebec, Canada; h Research Institute of the McGill University Health Centre, Montreal, Quebec, Canada

**Keywords:** Air pollution, Sector, Environmental Health, Environmental epidemiology, Neurological diseases, Mortality

## Abstract

**Background:**

Improving air quality requires addressing sector-specific air pollution (SSAP). This study examined the relationship between long-term SSAP and Alzheimer’s disease and dementia mortality in Canada, and whether associations were modified by neighborhood greenness, educational attainment, and material deprivation.

**Methods:**

We used data from the 2006 Canadian Census Health and Environment Cohort with mortality follow-up through 2019, linked to the Canadian Vital Statistics—Death database. Annual exposures to ambient air pollutants (i.e., PM_2.5_, SO_2_, NO_2_, and O_3_) from multiple sectors were estimated using the Global Environmental Multiscale-Modelling Air Quality and Chemistry model (10 km resolution) with sector-specific contributions anchored to 2015 emissions profiles. Quantile g-computation models were used to estimate hazard ratios (HRs) for Alzheimer’s disease and dementia per quartile increase in SSAP.

**Results:**

Alzheimer’s disease mortality was most strongly associated with SSAP from residential fuel combustion (RES: HR = 1.29; 95% CI: 1.16, 1.43), and was also positively associated with emissions from on-road transportation (HR = 1.22; 95% CI: 1.12, 1.32), ore and mineral industries (ORE: HR = 1.17, 95% CI: 1.10, 1.24), air–marine–rail transportation (HR = 1.12; 95% CI: 1.06, 1.18), and manufacturing (MAN: HR = 1.06; 95% CI: 1.01, 1.11), while inverse associations were observed for the oil and gas sector (HR = 0.85; 95% CI: 0.81, 0.88). Dementia mortality was positively associated with oil and gas (HR = 1.06; 95% CI: 1.03, 1.09), and inversely associated with air–marine–rail transportation (HR = 0.88; 95% CI: 0.85, 0.92) and ORE (HR = 0.89; 95% CI: 0.85, 0.92). Associations were generally stronger in lower greenness areas and among individuals with lower educational attainment, although heterogeneity by sector was observed.

**Conclusion:**

SSAP mixtures were associated with Alzheimer’s disease and dementia mortality in Canada. The direction and magnitude of associations varied by sector and by environmental and sociodemographic context, supporting the value of targeted, sector-specific mitigation strategies to reduce neurodegenerative mortality risk.

**What this study adds**

Although previous studies have linked ambient air pollution to neurological disease, few have investigated SSAP exposures and how associations vary across social and environmental contexts. We studied over 3 million adults in the Canadian Census Health and Environment Cohort (2006–2019), linking modeled SSAP, including PM_2.5_, NO_2_, SO_2_, and O_3_, to mortality. We observed higher Alzheimer’s disease mortality associated with exposure to RES, ONRD, ORE, and MAN emissions, and higher dementia mortality associated with oil and gas. Associations were modified by greenness, education, and material deprivation, highlighting populations most vulnerable to SSAP.

## Introduction

Alzheimer’s disease (AD) and other dementias are major global public health concerns and account for the largest and fastest growing portion of neurological disability and mortality worldwide. According to the Global Burden of Disease Initiative, dementia alone accounted for approximately 9.0 million deaths worldwide in 2016, and represents one of the leading causes of death in older adults.^1^ With population aging, dementia-related mortality is projected to increase substantially over coming decades, imposing growing burdens on health systems, long-term care infrastructure, and informal caregivers. Although dementia is typically studied as incidence, mortality is a clinically meaningful and policy-relevant endpoint because neurodegenerative conditions are progressive, contribute to long-term care demand, and are often under-ascertained in cause-of-death coding.

A growing body of evidence links long-term exposure to ambient air pollution (AP) with increased risk of cognitive decline, dementia, and AD.^2,3^ Fine particulate matter (PM_2.5_), nitrogen dioxide (NO_2_), sulfur dioxide (SO_2_), and ozone (O_3_) have each been implicated through biological mechanisms involving oxidative stress, neuroinflammation, cerebrovascular dysfunction, and disruption of the blood–brain barrier.^4–10^ PM_2.5_ in particular may penetrate the central nervous system directly or indirectly via systemic inflammation, while NO_2_ has been associated with dementia incidence and mortality, and O_3_ and SO_2_ with accelerated cognitive decline.^7–9^

Most prior studies have examined total ambient AP or traffic-related exposures, with limited attention to sector-specific air pollution (SSAP) arising from distinct emission sources such as transportation, industry, power generation, and residential fuel combustion. Emission sectors differ systematically in pollutant composition, chemical reactivity, spatial distribution, exposed populations, and regulatory controllability, suggesting that SSAP mixture analyses may provide information directly relevant to mitigation strategies and air-quality management.

Accordingly, this study examines long-term exposure to SSAP mixtures of PM_2.5_, NO_2_, SO_2_, and O_3_ and their associations with AD and dementia mortality in Canada. We further assess whether these associations differ by neighborhood greenness, educational attainment, and material deprivation as indicators of environmental buffering and social vulnerability. Identifying high-impact emission sectors and vulnerable contexts may inform targeted interventions to reduce the growing burden of neurodegenerative mortality.

## Methods

### Study cohort

The 2006 Canadian Census Health and Environment Cohort (CanCHEC) is a population-based linked cohort with mortality follow-up from May 16 2006 to December 31 2019. Detailed cohort linkage methods have been described previously^11^ and are summarized in Supplementary Content—Methods (S1), https://links.lww.com/EE/A413.

### Outcome definition

Mortality outcomes were defined using the Canadian Vital Statistics—Death Database, with causes of death coded according to the International Classification of Diseases, 10th revision (ICD-10).^12^ AD mortality was identified using code G30, and dementia mortality was identified using codes F00–F03.

AD represents a specific neuropathologic diagnosis, whereas dementia is a broader clinical syndrome that may include AD as an underlying cause. Diagnostic coding practices vary across jurisdictions and over time, and AD may be underreported when dementia is recorded as the primary cause of death. To maximize sensitivity and consistency with prior population-based studies, we defined events as deaths for which AD or dementia appeared as either the underlying or a contributing cause of death.

We analyzed AD and dementia mortality as distinct outcomes to explore potentially different epidemiologic patterns and sector-specific associationswhile acknowledging their partial diagnostic overlap. Any resulting outcome misclassification is expected to be largely nondifferential with respect to AP exposure and, therefore, likely to bias effect estimates toward the null.

### Exposure data

Estimates of sector-specific contributions to ambient AP concentrations were obtained from the Global Environmental Multiscale-Modelling Air Quality and Chemistry (GEM-MACH) model, which integrates emissions data for mobile, point, and area sources with meteorological data from a regional atmospheric model to simulate ambient concentrations of selected pollutants at a 10 km horizontal resolution. GEM-MACH is widely used for air quality forecasting and research, demonstrating robustness across a wide range of applications.^13–16^

SSAP concentrations were simulated using GEM-MACH for 2015, the year for which detailed national emissions inventories and source apportionment were available. For the full follow-up period (2006–2019), annual SSAP concentrations were derived by applying the 2015 sectoral contribution profiles to annual total pollutant concentration fields. This approach preserves temporal variation in overall AP while assuming stability in the relative contribution of emission sectors over time.

To evaluate the robustness of this assumption, we compared sector simulations for 2015 and 2019, examined historical national emissions trends, and conducted sensitivity analyses using alternative exposure scaling scenarios for periods in which SSAP concentrations differed by more than 10% from 2015 levels (see Sensitivity analyses). These analyses demonstrated minimal impact on hazard ratio (HR) estimates, supporting the temporal stability of the main findings.

Contributions to ambient AP were categorized into eight sectors based on source type:

- On-road transportation (ONRD; exhaust, evaporative, and tire/brake wear emissions from light and heavy-duty vehicles);
- Off-road transportation (OFRD; mobile equipment used in mining, construction, agriculture, and recreational activities);
- Air–marine–rail transportation (AMR; emissions from aircraft, marine vessels, and rail operations);
- Ore and mineral industries (ORE; including cement manufacturing and nonferrous refining and smelting);
- Electric power generation (EPG; coal-fired generation);
- Manufacturing (MAN; chemical and pulp and paper industries);
- Oil and gas (OAG; including upstream extraction and downstream refining, processing, and distribution); and,
- Residential fuel combustion (RES; fossil fuel and wood-burning for heating).

Emissions inputs were derived from Environment and Climate Change Canada’s Air Pollution Emissions Inventory, based on a combination of calculated and industry-reported data. The data were spatially allocated using the US EPA Sparse Matrix Operator Kernel Emissions (SMOKE, version 3.7) model, and incorporated into GEM-MACH version 2.3.1. Individual-level exposures were assigned annually using residential postal codes linked to geographic coordinates via Statistics Canada’s Postal Code Conversion File Plus (PCCF+). Detailed geocoding procedures are described in Supplementary Content—Methods (S2), https://links.lww.com/EE/A413. Additional details on sectoral attribution are provided in Supplementary Content—Methods (S3), https://links.lww.com/EE/A413.

### Statistical analysis

The CanCHEC cohort was followed from 2006 to 2019. Exposures estimated by the GEM-MACH model were linked to the cohort based on residential postal codes. We applied single-variable Cox proportional hazard models to assess associations between each covariate, and AD and dementia mortality. These models were used for descriptive purposes to characterize baseline sociodemographic and geographic risk patterns and to contextualize results from fully adjusted mixture models, rather than to support causal inference.

#### Time scale

We used attained age as the time scale in all Cox proportional hazards models, with individuals entering the risk set at their age on census day (16 May 2006) and followed until death or censoring at the end of follow-up (31 December 2019). The baseline hazard function was stratified by sex and immigration status.

#### Covariate selection

Covariates were selected *a priori* based on existing epidemiologic literature on dementia risk, known determinants of AP exposures, rather than through data-driven variable selection procedures. Fully adjusted models included individual- and area-level covariates: Indigenous identity, racialized group, marital status, educational attainment, occupational group, labor force status, neighborhood income quintile, urban form, Census Metropolitan Area or Census Agglomeration size, airshed, and four dimensions of the Canadian Marginalization Index (material deprivation, economic dependency, ethnic concentration, and residential instability).

#### Quantile g-computation models

To assess the joint effect of multiple pollutants within each sector, we employed quantile g-computation models to estimate HRs and 95% CIs for time to death from AD or dementia per quartile increase in each sector-specific pollutant mixture (SO_2_, NO_2_, PM_2.5_, and O_3_).^17^ This method enables estimation of the combined effect of correlated exposures while accommodating pollutants with opposing directional contributions within the mixture, without requiring specification of interaction terms or independent effects for individual pollutants.

Models were implemented using the *qgcomp* package^18^ in R version 4.2.3 (R Foundation for Statistical Computing, Vienna, Austria) and adjusted for the covariates listed above. Pollutant concentrations were categorized into quartiles based on their empirical distributions in the study cohort. Models were estimated without bootstrapping (noboot option), with CIs obtained directly from model estimates. Within each model, relative weights were calculated to reflect the proportional contribution of each pollutant to the overall mixture effect. Quantile g-computation addresses collinearity among correlated pollutants by transforming exposures into quantiles and estimating a single joint effect index. Mixture weights may be positive or negative depending on the direction of association and are constrained to sum to one within each direction. These weights do not represent independent effect estimates or statistical significance of individual pollutants, but instead indicate their relative contribution to the joint mixture effect, conditional on the correlation structure of the exposures. Accordingly, inverse weights or HRs should not be interpreted as evidence of protective effects. Further details on quantile g-computation are provided by Keil et al.^18^

#### Effect modification

Effect modification by neighborhood greenness, educational attainment, and material deprivation was evaluated using stratified quantile g-computation Cox models, and interpreted on the multiplicative scale using HRs. These analyses were intended to characterize heterogeneity in associations across social and environmental contexts rather than to estimate formal interaction parameters.

Neighborhood greenness was measured using the Normalized Difference Vegetation Index (NDVI), dichotomized at 0.5 within a 250-m buffer of each residence.^19,20^ Educational attainment was classified as less than high school versus high school or higher at the individual level. Material deprivation was defined using the the Canadian Marginalization Index, comparing the two most deprived quintiles with the remaining population.^21–26^

### Sensitivity analyses

As an additional sensitivity analysis, we reestimated all primary models restricting outcomes to deaths in which AD (ICD-10 G30) or dementia (ICD-10 F00–F03) was coded as the underlying cause of death only, and compared results with those obtained using our primary definition based on underlying or contributing causes. This analysis was conducted to assess the potential impact of diagnostic overlap and outcome misclassification associated with the inclusion of contributing causes of death.

#### Smoking

The 2006 CanCHEC dataset does not include individual-level smoking data. However, prior studies using similar Canadian cohorts have evaluated this limitation. Cakmak et al. used the Canadian Community Health Survey to indirectly adjust for smoking in AP–mortality models and reported minimal impact on estimates for lung cancer and cardiorespiratory mortality.^27^ Similarly, Weichenthal et al. observed little change in the association between ultrafine particle exposure and brain cancer incidence after indirect smoking adjustment.^28^ Based on these findings, we did not conduct indirect smoking adjustment in the present analysis.

#### Ethics

This study underwent ethics review by the Research Ethics Board at Health Canada. For each cycle of data, additional ethics approval was obtained by Statistics Canada. All data are kept strictly confidential under the Statistics Act, and results are published only in aggregated form.

## Results

### SSAP contributions

Across Canada, SSAP contributions to ambient SO_2_, NO_2_, PM_2.5_, and O_3_ varied markedly (Table S2, https://links.lww.com/EE/A413). RES produced the highest mean PM_2.5_ (1.37 µg/m^3^), while EPG contributed the least (0.06 µg/m^3^). ONRD was the primary source of NO_2_ (2.90 ppbv), compared with only 0.09 ppbv from EPG. ORE contributed most to SO_2_ (0.43 ppbv), whereas OFRD was negligible (<0.01 ppbv). For O_3_, ONRD was the largest contributor (0.75 ppbv), while the ORE and RES each contributed 0.04 ppbv.

### Single-variable Cox models (covariate analysis)

Cox proportional hazards models revealed significant associations between sociodemographic and geographic covariates, and mortality outcomes (Table S3, https://links.lww.com/EE/A413). Males had a lower hazard of AD mortality, but elevated hazards for dementia mortality compared with females. Higher neighborhood income quintile and educational attainment were consistently associated with lower mortality risk. Marital status also influenced outcomes, with separated individuals having the lowest AD mortality risk but elevated risks for dementia. Unemployment was associated with increased risk across all outcomes. Nonimmigrant status was linked to higher mortality risks, while racialized group status was associated with lower risk. Indigenous identity was positively associated with dementia. Greater residential instability and material deprivation were linked to elevated AD mortality, whereas the associations for neighborhood ethno-cultural concentration and economic dependency varied. Larger population centers showed higher dementia-related mortality compared with rural areas. Regional variations were also evident, with higher hazards observed in airsheds (e.g., Southern Atlantic region).

### Quantile g-computation Cox models

Primary analyses were based on deaths in which AD or dementia appeared as either the underlying or a contributing cause of death, consistent with the outcome definition described in the Methods.

### AD mortality

**Figure 1.**
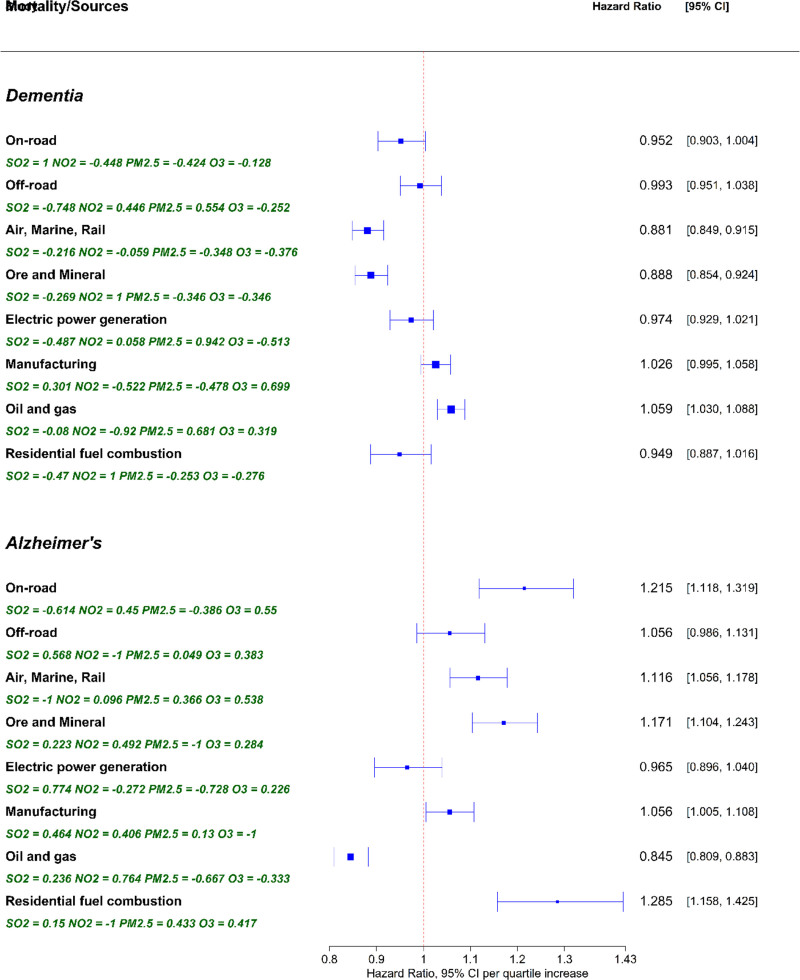
Mortality risks from dementia and Alzheimer’s disease associated with long-term exposure to sector-specific air pollution in Canada. Joint effects are expressed as hazard ratios (HRs) per interquartile range (IQR) increase in all pollutants within each mixture. Pollutants differ in concentration, chemical composition, and toxicity across sectors. The joint effects of SO_2_, NO_2_, PM_2_._5_, and O_3_ were estimated using quantile g-computation models for cause-specific mortality. Relative weights indicate the proportional contribution of each pollutant to the mixture effect. Negative weights sum to 1, as do positive weights, but the total contribution of positive versus negative weights is not assumed to be equal. Weights are only comparable within the same direction (i.e., positive or negative).

AD was the underlying cause in 8,055 deaths and a contributing cause in 4,575 (Table 1). Significant positive associations were found for several sectors (Figure 1). RES showed the strongest risk [HR = 1.285; 95% CI: 1.158, 1.425], with contributions from PM_2.5_ (43.3%), O_3_ (41.7%), and SO_2_ (15.0%). Elevated risks were also linked to ONRD (HR = 1.215; 95% CI: 1.118, 1.319), ORE (HR = 1.171; 95% CI: 1.104, 1.243), AMR (HR = 1.116; 95% CI: 1.056, 1.178), and MAN (HR = 1.056; 95% CI: 1.005, 1.108) SSAP exposure. By contrast, OAG concentrations were inversely associated with AD mortality (HR = 0.845; 95% CI: 0.809, 0.883), mainly due to negative contributions from PM_2.5_ (66.7%) and O_3_ (33.3%). OFRD and EPG showed no significant associations.

**Table 1.** Person-years and deaths counts for causes of interest, for underlying and contributing causes of death

Cause of death	Person-years (n)^a^	Underlying cause of death	Underlying or contributing cause of death
Alzheimer’s disease	36,112,640	8,055	12,630
Dementia	36,112,640	19,970	44,460
Parkinson’s	36,112,640	3,575	6,690

a Person-years follow-up.

### Dementia mortality

Dementia was the underlying cause of 11,585 deaths and a contributing cause in 31,900 (Table 1). SSAP from OAG were positively associated with dementia mortality (HR = 1.059; 95% CI: 1.030, 1.088), with contributions from PM_2.5_ (68.1%) and O_3_ (31.9%). In contrast, inverse associations were observed for AMR (HR = 0.881; 95% CI: 0.849, 0.915) and ORE (HR = 0.888; 95% CI: 0.854, 0.924) SSAP, with negative contributions largely from PM_2.5_, O_3_, and SO_2_. No significant associations were observed for ONRD, OFRD, EPG, MAN, or RES. Results from single-pollutant models were consistent in the direction of effects (Table S4, https://links.lww.com/EE/A413).

### Sensitivity analyses

When outcomes were restricted to deaths in which AD or dementia was recorded as the underlying cause only, AD and dementia diagnoses were mutually exclusive by design. Among deaths with dementia as the underlying cause (n = 19,970), 0.34% (n = 70) also listed AD as a contributing cause, while among deaths with AD as the underlying cause (n = 8,055), 6.8% (n = 550) listed dementia as a contributing cause. Associations between SSAP and dementia mortality remained directionally consistent with primary analyses, but were modestly attenuated when contributing causes were excluded. 95% CIs overlapped across outcome definitions, providing evidence there are no statistically meaningful differences in effect estimates.

To assess the robustness of findings to assumptions regarding the temporal stability of SSAP, we conducted additional sensitivity analyses applying alternative scaling scenarios to annual sector-specific contributions. Sector-specific concentrations of PM_2.5_, NO_2_, and SO_2_ were rescaled for two historical periods (2006–2009 and 2010–2014) in which national emissions inventory data indicated that sectoral contributions differed by more than 10% from 2015 levels.

Across these scenarios, HR estimates for AD and dementia mortality were highly consistent with primary analyses. Absolute changes in HRs were generally <1% across sectors, with the largest observed difference for the OFRD sector (<0.85%). No sensitivity analysis resulted in a change in the direction or statistical significance of the main associations, and sector-specific patterns and mixture weights were preserved, indicating that the primary findings were robust to plausible temporal variation in sector-specific emissions over the study period. Estimates using underlying-cause-only definitions were modestly attenuated relative to the primary definition but remained directionally consistent with overlapping CIs, and sector-specific patterns and mixture weights were preserved.

### Effect modification

#### Greenness

Results suggested NDVI modified the associations between SSAP and AD mortality (Table 2). Regarding AD mortality, positive associations were stronger and statistically significant in low-NDVI areas, while they were weaker and often null in high NDVI areas. This effect modification was consistent across multiple sectors. For instance, SSAP from ONRD was associated with significantly elevated AD mortality risk in low-NDVI areas (HR = 1.228; 95% CI: 1.108, 1.361), but not in high NDVI areas (HR = 1.039; 95% CI: 0.919, 1.174). Similar trends were observed for the AMR, OFRD, and ORE.

**Table 2.** Associations between sector-specific air pollution (SSAP) mixtures and Alzheimer’s disease mortality, stratified by neighborhood greenness (NDVI), educational attainment, and material deprivation

	HR (95% CI)	Air pollutant weights			
		SO_2_	NO_2_	PM_2.5_	O_3_
Low NDVI (<0.5)					
ONRD	1.228 (1.108, 1.361)	−1.000	0.433	0.054	0.513
OFRD	1.092 (1.003, 1.189)	0.105	−1.000	0.515	0.380
AMR	1.181 (1.108, 1.258)	−1.000	0.104	0.231	0.665
ORE	1.285 (1.196, 1.381)	0.110	0.438	0.017	0.435
EPG	1.003 (0.914, 1.100)	0.672	−0.271	−0.729	0.328
MAN	1.024 (0.966, 1.086)	0.125	0.875	−0.403	−0.597
OAG	0.847 (0.801, 0.894)	−0.230	1.000	−0.473	−0.297
RES	1.322 (1.177, 1.485)	0.089	0.040	0.513	0.359
High NDVI (≥0.5)					
ONRD	1.039 (0.919, 1.174)	0.728	−0.906	0.727	−0.094
OFRD	0.969 (0.874, 1.075)	0.861	−0.356	−0.644	0.139
AMR	1.054 (0.956, 1.161)	−0.212	−0.788	0.077	0.923
ORE	1.036 (0.940, 1.143)	0.209	0.703	−1.000	0.087
EPG	0.996 (0.873, 1.136)	0.838	−0.451	−0.549	0.162
MAN	1.040 (0.956, 1.131)	0.042	−0.565	0.958	−0.435
OAG	0.835 (0.775, 0.900)	−0.251	1.000	−0.357	−0.392
RES	1.239 (1.036, 1.480)	−1.000	0.139	0.602	0.260
Low education attainment (<high school)					
ONRD	1.108 (0.988, 1.243)	0.550	−1.000	0.091	0.359
OFRD	1.114 (1.015, 1.222)	0.739	−0.520	−0.480	0.261
AMR	1.181 (1.093, 1.278)	0.340	−1.000	0.087	0.573
ORE	1.102 (1.010, 1.202)	0.431	−1.000	0.053	0.516
EPG	1.070 (0.965, 1.186)	0.529	−0.225	−0.775	0.471
MAN	1.111 (1.032, 1.196)	0.332	0.258	0.410	−1.000
OAG	0.806 (0.752, 0.864)	−0.266	1.000	−0.414	−0.320
RES	1.121 (0.961, 1.308)	−0.337	−0.663	0.901	0.099
High education attainment (high school diploma or higher)					
ONRD	1.112 (0.994, 1.244)	−1.000	0.101	0.239	0.660
OFRD	1.055 (0.963, 1.156)	0.242	−1.000	0.358	0.400
AMR	1.053 (0.979, 1.132)	−1.000	0.102	0.314	0.584
ORE	1.186 (1.092, 1.287)	0.259	0.492	−1.000	0.249
EPG	0.918 (0.825, 1.022)	1.000	−0.268	−0.631	−0.101
MAN	0.977 (0.915, 1.042)	0.986	−0.288	0.014	−0.712
OAG	0.860 (0.812, 0.910)	−0.006	−0.163	−0.450	−0.380
RES	1.224 (1.077, 1.392)	0.228	0.056	0.360	0.355
Low material deprivation (top two quintiles of CMSDI)					
ONRD	1.278 (1.136, 1.438)	0.349	0.340	−1.000	0.311
OFRD	1.124 (1.018, 1.241)	0.380	0.306	−1.000	0.314
AMR	1.159 (1.056, 1.272)	0.258	−1.000	0.027	0.715
ORE	1.045 (0.959, 1.138)	0.579	0.005	−1.000	0.416
EPG	1.014 (0.919, 1.118)	0.638	0.038	−1.000	0.324
MAN	1.088 (1.008, 1.175)	0.329	0.037	0.634	−1.000
OAG	0.816 (0.762, 0.874)	−0.047	1.000	−0.603	−0.349
RES	1.259 (1.080, 1.468)	0.264	−1.000	0.466	0.270
High material deprivation (bottom two quintiles of CMSDI)					
ONRD	1.132 (0.973, 1.316)	−0.048	0.503	−0.952	0.497
OFRD	0.996 (0.884, 1.122)	0.599	−1.000	0.089	0.312
AMR	0.990 (0.905, 1.084)	−0.957	−0.043	0.824	0.176
ORE	1.278 (1.136, 1.439)	−1.000	0.412	0.235	0.352
EPG	0.858 (0.739, 0.997)	1.000	−0.645	−0.093	−0.262
MAN	0.938 (0.859, 1.025)	1.000	−0.375	−0.037	−0.587
OAG	0.942 (0.872, 1.018)	−0.370	1.000	−0.417	−0.212
RES	1.028 (0.861, 1.227)	0.624	−1.000	0.267	0.109

AMR indicates air–marine–rail transportation; CI, confidence interval; CMSDI, Canadian Marginalization Social Deprivation Index; EPG, electric power generation; HR, hazard ratio; MAN, manufacturing; NDVI, normalized difference vegetation index; NO_2,_ nitrogen dioxide; O_3_, ozone; OAG, oil and gas; OFRD, off-road transportation; ONRD, on-road transportation; ORE, ore and mineral industries; PM_2.5_, particulate matter ≤2.5 µm in diameter; RES, residential fuel combustion; SO_2,_ sulphur dioxide.

#### Educational attainment

Educational attainment also modified the relationship between AP exposure and AD mortality (Table 2).

For AD mortality, stronger and statistically significant associations were observed among individuals with lower educational attainment. For example, exposure to the AMR SSAP was associated with increased AD mortality in the lower education group (HR = 1.181; 95% CI: 1.093, 1.278), but not in the higher education group (HR = 1.053; 95% CI: 0.979, 1.132). Similar trends were noted for MAN and OFRD sectors. However, for RES, a stronger association was observed among individuals with higher educational attainment (HR = 1.224; 95% CI: 1.077, 1.392), compared with those with lower educational attainment (HR = 1.121; 95% CI: 0.961, 1.308).

#### Material deprivation

Material deprivation significantly modified the effects of SSAP on AD mortality (Table 2).

Stronger and statistically significant associations were generally observed in low deprivation areas across several sectors, including AMR, MAN, ONRD, OFRD, and RES. For example, ONRD AP exposure was associated with AD mortality in the low deprivation group (HR = 1.278; 95% CI: 1.136, 1.438), while the association was weaker yet nonsignificant in the high deprivation group (HR = 1.132; 95% CI: 0.973, 1.316). However, in some instances, elevated risks were observed in the high deprivation group. For instance, AP exposure from the ORE sector was associated with significantly higher AD mortality in high deprivation areas (HR = 1.278; 95% CI: 1.136, 1.439) compared with low deprivation areas (HR = 1.045; 95% CI: 0.959, 1.138). Similarly, exposure to the EPG sector showed a significant inverse association in the high deprivation group (HR = 0.858; 95% CI: 0.739, 0.997), but not in the low deprivation group (HR = 1.014; 95% CI: 0.919, 1.118). Lastly, the OAG sector was inversely related in the low deprivation group (HR = 0.816; 95% CI: 0.762, 0.874), but this association was attenuated and nonsignificant in high deprivation areas (HR = 0.942; 95% CI: 0.872, 1.018).

## Discussion

In this national cohort study, long-term exposure to SSAP mixtures was associated with mortality from AD and dementia, with substantial heterogeneity across emission sectors and social and environmental contexts. RES, ONRD, AMR, ORE, and MAN sectors were associated with higher AD mortality, whereas OAG mixtures were associated with higher dementia mortality. Several associations differed according to neighborhood greenness, educational attainment, and material deprivation, with several associations stronger in low-greenness areas and among individuals with lower educational attainment, although patterns varied by sector. These findings indicate that both emission source characteristics and contextual vulnerability shape neurodegenerative mortality risk.

### SSAP exposure and mortality from dementia and AD

While numerous studies have examined the relationship between AP and mortality from AD and dementia, few have focused specifically on SSAP and/or used multipollutant models. Our study identified significant associations between SSAP mixtures (SO_2_, NO_2_, PM_2.5_, and O_3_) and mortality from AD and dementia, with heterogeneity by sector and outcome.

In quantile g-computation, mixture weights reflect the relative contribution of each pollutant to the overall joint effect estimate, conditional on the correlation structure and scaling of the exposures, and do not represent independent or causal effects. For example, for RES, PM_2.5_ and O_3_ accounted for approximately 43% and 42% of the positive mixture index, respectively, indicating that these pollutants contributed most strongly to the estimated joint association for that sector. Negative weights reflect contributions opposite to the overall mixture index and should not be interpreted as biologically protective.

Inverse associations observed for some sectors and outcomes should not be interpreted as protective effects and may reflect residual confounding, differential diagnostic or reporting practices across regions, exposure measurement error, or limitations in sector attribution over time. HRs below 1.0 and negative mixture weights should therefore be interpreted cautiously as features of the statistical mixture framework rather than evidence of benefit.

Increased dementia mortality risk was associated with exposure to the OAG sector, whereas an inverse association was noted with AMR, and ORE sectors. In contrast, AD mortality risk increased with AP exposure from ONRD, AMR, ORE, MAN, and RES, but an inverse risk for the OAG sector. No significant associations were observed between either AD or dementia mortality in relation to the OFRD or EPG sectors.

These findings do not imply that dementia and AD should be prioritized over other pollution-related health outcomes in air-quality regulation. Rather, sector-specific evidence for neurodegenerative mortality complements existing multioutcome assessments by identifying emission sources that may disproportionately contribute to long-term neurological burden. Because regulatory interventions are typically implemented at the sector level (e.g., transportation standards, residential heating policies, industrial emissions controls), SSAP analyses provide actionable information that pollutant-based models alone cannot offer. Sensitivity analyses examining alternative temporal scaling of sector-specific emissions demonstrated minimal impact on HRs, supporting the robustness of the primary findings (see Results: Sensitivity analyses).

### AP contributions to mortality risks

#### Differential mortality risks for AD versus dementia

Our findings underscore the importance of evaluating SSAP mixtures rather than individual pollutants. Each sector emits a distinct mixture, and health effects vary by composition and outcome. For example, O_3_ substantially contributed to AD mortality from ONRD and AMR, consistent with studies linking long-term O_3_ exposure to neurological mortality, including Canadian regional differences.^29^

For ORE, NO_2_ was the dominant contributor, with smaller roles for O_3_ and SO_2_. MAN concentrations showed a more balanced influence of SO_2_ and NO_2_. As previously mentioned, negative PM_2.5_ contributions from ONRD and ORE sectors should not be interpreted as protective effects.

Results differ from a meta-analysis by Xie et al., which reported no significant associations between long-term NO_2_ or O_3_ exposure and AD risk,^30^ while no studies have specifically assessed SO_2_. Differences in copollutant mixtures, dispersion, and geography may explain discrepancies. For instance, NO_2_ from traffic often co-occurs with PM_2.5_ and black carbon in dense urban areas, whereas NO_2_ from the ORE sector arises in more industrial or remote regions. In contrast, RES associations with AD were primarily driven by PM_2.5_ and O_3_, aligning with prior meta-analyses linking PM_2.5_ to AD and dementia.^30–32^

OAG concentrations were inversely associated with AD mortality, driven by NO_2_ and SO_2_. This counterintuitive result may reflect unmeasured confounding, such as regional socioeconomic or healthcare differences, or a low-dose threshold effect given lower PM_2.5_ concentrations (0.105 µg/m^3^) compared with residential fuel combustion (1.367 µg/m^3^). At low levels, adverse effects may not manifest, and residual confounding may produce an apparent protective effect.

Future studies could consider aggregating gaseous pollutants (SO_2_, NO_2_, O_3_) across sectors while retaining sector-specific PM_2.5_ estimates. This approach may simplify interpretation by isolating the most compositionally distinct SSAP.

#### Patterns of mortality risk across social, environmental, and geographic contexts

Effect modification analyses were undertaken to evaluate differential vulnerability and exposure context rather than differences in intrinsic pollutant toxicity. Educational attainment and material deprivation capture dimensions of cognitive reserve, healthcare access, comorbidity burden, housing quality, and occupational exposure patterns, while neighborhood greenness may modify both exposure (e.g., pollution attenuation, heat mitigation) and susceptibility (e.g., stress reduction, physical activity, sleep quality). Heterogeneity across these dimensions therefore provides insight into populations and settings where SSAP may have the greatest health impact.

This study examined how individual and neighborhood factors shape both baseline mortality risk and susceptibility to SSAP across AD and dementia mortality. Our findings show these factors influence outcomes directly and as modifiers of AP effects. Higher education and residence in higher income neighborhoods were associated with lower mortality, consistent with prior work linking socioeconomic advantage to better access to healthcare, stable housing, and protective resources.^33,34^ In contrast, neighborhood material deprivation and residential instability were associated with higher AD mortality. Nonimmigrants also had higher mortality, aligning with evidence that immigrants to Canada often experience a “healthy immigrant effect” due to health screening at entry.^35,36^ Mortality risks were higher among Indigenous participants, while racialized group status was associated with lower AD and dementia mortality, which warrants further study given well-documented health inequities.^37^

Geography also influenced risks. Large urban centers had higher dementia mortality compared with non-Census Metropolitan Area/Census Agglomeration areas. Regionally, elevated mortality in the Southern Atlantic airshed may reflect exposure from EPG. These findings echo a study by Chen and colleagues that observed greater dementia risk among urban residents near major roads.^31^

Effect modification analyses showed stronger SSAP-related AD mortality among those with lower education, consistent with prior evidence linking low educational attainment to higher AD risk.^24^ Stronger associations with the AMR, MAN, and OFRD sectors suggests disproportionate exposure in industrial or high-traffic areas. Conversely, higher-educated individuals exposed to RES faced greater AD mortality risk, possibly reflecting wood-burning for heating in suburban or affluent rural areas.

Material deprivation further modified associations. Higher AD mortality was observed among highly deprived individuals exposed to the ORE SSAP, consistent with prior studies linking low socioeconomic status to dementia.^38–40^ In contrast, positive associations were observed in low deprivation areas exposed to the AMR, MAN, and RES SSAP, while highly deprived groups exposed to EPG SSAP showed negative associations. These contrasting patterns suggest deprivation modifies SSAP–AD associations by sector.

Residential greenness also influenced risk. Participants in low-NDVI areas had stronger associations between SSAP and AD mortality, consistent with studies showing greenness reduces neurodegenerative risk.^26,40^ Protective mechanisms may include reduced inflammation and oxidative stress, promotion of physical activity, cognitive engagement, and social interaction, as well as lower stress and improved sleep. NDVI is a proxy and effects likely vary by vegetation type, density, and context, warranting further study. Stronger modifying effects were observed for exposures from the transportation-related and ORE SSAP, which often overlap with low-greenness areas such as urban corridors or mining regions. Greenness may also provide broader cardiovascular and respiratory benefits by improving air quality, supporting activity, and reducing heat exposure.

#### Study strengths and limitations

This study has several strengths in addressing knowledge gaps. Previous research relied on single- or multipollutant models prone to multicollinearity, with most focusing on overall or traffic-related exposures rather than industrial or residential sectors.^41–43^ By contrast, we applied quantile g-computation to evaluate SSAP, reducing collinearity and clarifying each pollutant’s contribution. Exposure estimates were further refined using the GEM-MACH model, which incorporates meteorological and topographical data to simulate pollutant transport and transformation. Use of the CanCHEC also provided sociodemographic data, long-term follow-up, and sufficient statistical power to detect associations. Our analyses also examined effect modification by education, material deprivation, and residential greenness, allowing a more nuanced examination of environmental health inequities.

Several limitations should also be noted. SSAP contributions were derived from 2015 GEM-MACH simulations and extrapolated to 2006–2019. While comparisons with later simulations and exposure inventories showed stability, sectoral contributions likely varied in earlier years due to regulatory, technological, or economic changes. Assigning exposures by residential postal code may have also introduced misclassification, as this approach does not account for mobility, indoor exposures, or local hotspots within the 10 km grid. The optimal spatial resolution for SSAP assessment is not well established and likely varies by emission sector, depending on pollutant composition, chemical reactivity, emission height, and atmospheric transport processes. Although finer spatial resolutions may be advantageous for attributing sources of specific pollutants, they would not necessarily improve individual-level exposure accuracy and could increase exposure misclassification in the presence of residential mobility and daily activity patterns. The use of a 10 × 10 km grid in this study reflects constraints of the GEM-MACH chemical transport model, Environment and Climate Change Canada’s Air Pollutant Emissions Inventory, and the US Environmental Protection Agency’s Sparse Matrix Operator Kernel Emissions (SMOKE) model. As such, some degree of spatial averaging is unavoidable, particularly for sectors with highly localized emissions. The attenuation observed when restricting outcomes to underlying cause of death only is consistent with nondifferential outcome misclassification when contributing causes are included and supports the robustness of our primary findings. Canada’s relatively low pollutant levels may also limit the detection of strong associations, and modeled estimates remain subject to inventory uncertainties. Finally, our analysis focused on single-sector mixtures, whereas real-world exposures reflect overlapping sectors with potential additive or synergistic effects. As SSAP varies spatially—for example, oil/gas in younger, affluent regions versus traffic in older, urban populations—future research should integrate high-resolution spatial and temporal data to better capture cross-sector exposures. Given the large number of sector–outcome–modifier comparisons evaluated, observed heterogeneity patterns should be interpreted cautiously and prioritized for replication in independent populations.

Mortality analyses estimated cause-specific hazards, with deaths from other causes treated as censored. Although competing risks may influence absolute risks, they are unlikely to materially affect relative effect estimates under nondifferential exposure misclassification. Sensitivity analyses applying alternative temporal scaling of sector-specific emissions demonstrated minimal impact on effect estimates, supporting the robustness of the observed associations to plausible variation in sectoral emission profiles over time.

## Conclusion

This study identified increased risks of AD and dementia mortality associated with long-term exposure to SSAP mixtures in Canada. Exposure from ONRD, RES, AMR, MAN, and ORE sectors were associated with higher AD mortality, while OAG was associated with higher dementia mortality. Several associations varied by socioeconomic and environmental context, indicating that SSAP-related health impacts depend not only on emission source characteristics but also on broader social and environmental vulnerability. These findings underscore the importance of evaluating SSAP and support targeted mitigation strategies—particularly in socioeconomically disadvantaged or low-greenness settings—to reduce the growing burden of neurodegenerative disease.

## Conflicts of Interest Statement

S.C. reports receiving funding from Health Canada as a Principal Investigator and is employed by Health Canada. K.M., C.H., A.F., and M.J. are also employed by Health Canada, and T.O. and M.T. work for Statistics Canada. The authors declare they have no known competing financial interests or personal relationships that could have appeared to influence the work reported in this paper.

## Acknowledgments

We would like to thank Health Canada for its support for this study, Statistics Canada for help accessing census and socioeconomic data, and Environment and Climate Change Canada (ECCC) for sector-specific data. We would also like to thank Nick Charman at Health Canada for his assistance with the sector contribution scaling factors used in the sensitivity analysis.

## Supplementary Material
