# Supplementary material for: Sector-specific Long-term Associations Between Transportation, Industrial, and Residential Combustion Air Pollutant Mixtures (PM2.5, SO2, NO2, O3) and Neurological Disease-related Mortality in Canada
Source: Environ Epidemiol. 2026 Mar 19;10(2):e467. doi: 10.1097/EE9.0000000000000467 (PMC13004229; doi:10.1097/EE9.0000000000000467)
Supplement: Supplementary file 1 [file ee9-10-e467-s001.pdf]

Supplementary Content – Methods (S1)

The 2006 Canadian Census Health and Environment Cohorts (CanCHEC) is a population-based linked dataset incorporating mortality data (e.g., date, cause of death) and annual residential postal codes for environmental exposure assignment. This dataset uses Statistics Canada's Social Data Linkage Environment (SDLE) to link files via the Derived Record Depository (DRD), a dynamic relational database containing basic personal identifiers. Additional information on the linkage and cohort is described elsewhere.[11] For this study, mortality follow-up started on census day (i.e., May 16, 2006) and ended on December 31, 2019. During this period, 586,300 deaths were recorded, and causes of death were coded using the International Classification of Diseases, 10th Revision (ICD-10).[12] This study considered AD (i.e., ICD-10 code: G30); and, dementia (i.e., ICD-10 codes: F00-F03) mortality.

Supplementary Content – Methods (S2)

Individual-level exposures were assigned to CanCHEC participants, on a time-varying annual basis, using latitude and longitude coordinates from residential postal codes obtained from either an annual tax file or imputed full (or partial) postal code where gaps existed. These postal codes were geocoded using Statistics Canada's Postal Code Conversion File Plus (PCCF+),[1] which maps them to standard geographic areas (e.g., dissemination areas or census tracts) and assigns coordinates based on the centroids of the representative point (e.g. block face, dissemination block, or dissemination area). This approach captures both temporal variation in pollutant concentrations and spatial variation in residential locations over time.

Supplementary Content – Methods (S3)

We first compared sectoral contributions to ambient pollutant contributions from simulations with GEM-MACH model for the years 2015 and 2019. Sector descriptions are provided in Table S1. For 2015

and 2019, relative sector contributions to ambient air pollutant concentrations were calculated by comparing pollutant concentrations between two modelling scenarios: (1) a base case that included all air pollution (AP) sectors, and (2) a scenario in which exposures from the sector of interest were removed. The percentage contributions to pollutant concentrations (NO_2, O_3, $PM_{2.5}$, SO_2) were found to be stable across 2015 and 2019 for the selected sectors. To estimate annual sector-specific air pollution (SSAP) concentrations over the full exposure window from 2006 to 2019, the 2015 sector-specific relative contribution estimates were applied to observed annual mean concentrations from the National Air Pollution Surveillance (NAPS) monitoring network from 2006 to 2019. Although we assumed relative sectoral contributions to ambient AP were consistent throughout the study period, the magnitude of exposure was adjusted to reflect measured ambient concentrations for each pollutant of interest. As ambient concentrations for pollutants were generally higher in the earlier years of the study, this adjustment augmented the sectoral exposure estimates for earlier years.

We further conducted a sensitivity analysis on the models for AD mortality using historical data from the National Pollutant Release Inventory (NPRI) and the Air Pollutant Emissions Inventory (APEI) to assess the potential influence of temporal changes in sector-specific contributions. For the sensitivity analysis, we divided the study period into three approximately equal intervals (2006–2009, 2010–2014, and 2015–2019) and scaled the sector-specific contributions to annual air pollutant concentrations relative to average emissions in each period, using 2015 as the reference year. Where sector-specific emissions differed by more than 10% from 2015 estimates, scaling factors were calculated as one-half of the difference between 2015 emissions and average reported emissions for each of three time periods. Analysis of the 2015 simulation results with the scaling factors applied showed a generally linear association between changes in NOx and $PM_{2.5}$ emissions and NO_2 and $PM_{2.5}$ concentrations, supporting our sensitivity assumption. We also calculated normalized scaling factors, by normalizing sectoral emissions to total emissions (all sectors in the inventory) for each period, which confirmed the primary

scaling factors used in the sensitivity analysis were conservative. The sensitivity analysis primarily adjusted the transportation and oil/gas sectors, reflecting their greater emissions variability across the study period. This conservative adjustment was chosen to reflect major temporal shifts in sector emissions without over-correcting for potential non-linearities between emissions and ambient concentrations. O_3 contributions were not scaled, as their ambient concentrations are strongly influenced by complex, non-linear atmospheric chemistry. involving multiple precursors.

Supplementary Content – Tables

Table S1. Air pollutant emissions inventory sector descriptions (Environment and Climate Change Canada, 2024)

Source/sector	Specific Sources within the Sector
**ONRD**	Includes exhaust emissions, evaporative emissions, and tire/brake wear emissions for all on-road light duty vehicles (LDV) and heavy duty vehicles (HDV).
On-road LDV	Exhaust emissions, evaporative emissions, and tire/brake wear emissions from vehicles under 3,856 kg, including cars, trucks, motorcycles; all fuel types (gasoline, diesel, propane, natural gas).
On-road HDV	Exhaust emissions, evaporative emissions, and tire/brake wear emissions from vehicles > 3,856 kg; all fuel types (gasoline, diesel, propane, natural gas).
**OFRD**	Exhaust emissions from off-road vehicles and mobile equipment using all fuel types (gasoline, diesel, propane, natural gas) in mining, construction, agriculture, commercial purposes, logging, railway maintenance, airport ground support, lawn and garden equipment, recreational vehicles.
**AMR**	The sum of air and marine and rail transportation sectors was modelled separately in this analysis.
Air transportation	Includes landing and take-off exhaust emissions for all piston and turbine aircraft (commercial, private, military). Does not include cruise emissions (released at high altitude above troposphere thus minimal impact on ground-level ambient population exposures).
Marine transportation	Includes exhaust emissions from all marine vessels operating within Canadian waters (domestic, international, fishing, military).
Rail transportation	Exhaust emissions from freight and passenger trains, including yard switching activities.
**ORE**	Includes multiple subsectors. Within this group of sectors, modelled separately were: cement manufacturing, non-ferrous refining and smelting.
Cement manufacturing	Includes emissions from entire process of cement production in rotary kilns, and preparation of concrete and ready-mix concrete, lime manufacture and concrete batching and products.

Non-ferrous refining and smelting	Includes primary copper and nickel production, lead ore crushing, concentrating and metallurgic processing, zinc metal production, and smaller non-ferrous refining and smelting sources (e.g., magnesium, cobalt and uranium industry processes).
**EPG**	Includes multiple subsectors. Within this group of sectors, coal-fired electric power generation was modelled separately.
Coal-fired electric power gen	Electric power generation from combustion of coal by utilities (publicly and privately owned) for commercial sales and/or private use.
**MAN**	Includes multiple subsectors. Within this group of sectors, modelled separately were: chemicals, pulp and Paper.
Chemicals	Product industries including fertilizer manufacturing, plastic resins, paints and varnishes, petrochemicals, inorganic chemicals, and pharmaceuticals.
Pulp and paper	Chemical, mechanical, recycling and semi-chemical mills, including the production of energy through the combustion of process waste products.
**OAG**	Overall oil/gas industry sector impacts were estimated by summing the two sub-sector model results modelled individually (i.e., upstream, downstream).
Upstream oil/gas	Refining and processing of crude oil to make fuels or other products (e.g., solvents, asphalt). Storage and distribution of refined petroleum products, natural gas distribution and liquid natural gas processing.
Downstream oil/gas	Drilling, testing and servicing of wells, conventional oil/gas production, in situ bitumen extraction and open-pit mining, oil sands upgrading, natural gas processing, crude oil transmission, natural gas transmission and storage.
**RES**	Combustion of fossil fuels used for space/water heating in residences. Includes residential wood combustion (i.e., home firewood burning).

Abbreviations: AMR, air-marine-rail transportation; EPG, electric power generation; MAN, manufacturing; OAG, oil and gas; OFRD, off-road transportation; ONRD, on-road transportation; ORE, ore and mineral industries; RES, residential fuel combustion.

Table S2. Mean, standard deviation and interquartile range (IQR) of air pollutants by source/sector.

Pollutant	Source	Mean	Std	IQR
SO_2 (ppb)	ONRD (LDVs, LDTs, motorcycles, and HDVs)	0.01	0.01	0.02
	OFRD (excluding air, marine and rail)	<0.01	<0.01	0.01
	AMR	0.02	0.06	0.01
	ORE	0.43	1.10	0.44
	EPG	0.04	0.11	0.02
	MAN	0.13	0.37	0.09
	OAG	0.12	0.27	0.08
	RES	<0.01	<0.01	0.01
NO_2 (ppb)	ONRD (LDVs, LDTs, motorcycles, and HDVs)	2.9	2.46	3.85
	OFRD (excluding air, marine and rail)	0.71	0.72	0.81
	AMR	0.56	0.59	0.52
	ORE	0.17	0.33	0.18
	EPG	0.09	0.20	0.08
	MAN	0.17	0.34	0.14
	OAG	0.17	0.44	0.07
	RES	0.42	0.40	0.51
$PM_{2.5}$ ($\mu g/m^3$)	ONRD (LDVs, LDTs, motorcycles, and HDVs)	0.49	0.39	0.59
	OFRD (excluding air, marine and rail)	0.29	0.26	0.31
	AMR	0.09	0.08	0.06
	ORE	0.50	0.59	0.51
	EPG	0.06	0.08	0.03
	MAN	0.14	0.15	0.12
	OAG	0.11	0.14	0.05
	RES	1.37	1.17	1.52
O_3 (ppb)	ONRD (LDVs, LDTs, motorcycles, and HDVs)	0.75	1.54	2.34
	OFRD (excluding air, marine and rail)	0.7	0.39	0.34
	AMR	0.41	0.62	0.28
	ORE	0.04	0.64	0.16
	EPG	0.11	0.20	0.05
	MAN	0.26	0.22	0.07
	OAG	0.54	1.07	0.05
	RES	0.04	0.08	0.19

Abbreviations: AMR, air-marine-rail transportation; CI, confidence interval; EPG, electric power generation; HDV, heavy-duty vehicles; HR, hazard ratio; IQR, interquartile range; LDV, light-duty vehicles; LDT, light-duty trucks; MAN, manufacturing; NDVI, normalized difference vegetation index; NO_2, nitrogen dioxide; O_3, ozone; OAG, oil and gas; OFRD, off-road transportation; ONRD, on-road transportation; ORE, ore and mineral industries; $PM_{2.5}$, particulate matter ≤2.5 micrometers in diameter; ppb, parts per billion; RES, residential fuel combustion; SO_2, sulphur dioxide; Std, standard deviation.

Table S3. HR (95% CI) of each of the selected covariates for time to deaths attributable from Alzheimer's disease and dementia from the single-variable Cox proportional hazard models.

Covariate	Sub-group	Person-Years (n)[a]	Alzheimer's Disease	Dementia
Sex	Female	18,870,760		
	Male	17,241,880	0.91 (0.87, 0.95)	1.66 (1.60, 1.72)
Neighbourhood income quintile	Lowest	6,109,120		
	Lower-middle	6,879,935	1.03 (0.97, 1.09)	0.94 (0.91, 0.98)
	Middle	7,381,300	1.01 (0.95, 1.08)	0.89 (0.85, 0.93)
	Upper-middle	7,737,320	0.97 (0.91, 1.05)	0.83 (0.79, 0.87)
	Upper	8,004,960	0.92 (0.85, 1.00)	0.75 (0.71, 0.79)
Educational attainment	<High school	7,178,305		
	High school	13,063,695	0.96 (0.91, 1.01)	0.93 (0.9, 0.96)
	Postsecondary non-university	7,139,625	0.80 (0.74, 0.87)	0.86 (0.82, 0.9)
	University degree	8,731,015	0.86 (0.80, 0.92)	0.80 (0.77, 0.84)
Marital status	Common law	4,533,120		
	Married	21,184,140	0.98 (0.85, 1.14)	1.18 (1.06, 1.31)
	Never married/not common law	5,281,785	1.09 (0.92, 1.28)	1.25 (1.11, 1.41)
	Separated	998,630	0.74 (0.57, 0.95)	1.25 (1.07, 1.46)
	Divorced	2,303,400	1.05 (0.88, 1.25)	1.38 (1.22, 1.56)
	Widowed	1,811,575	0.98 (0.84, 1.14)	1.24 (1.11, 1.38)
Employment status	Employed	23,966,395		
	Unemployed	1,267,850	1.68 (1.18, 2.40)	1.74 (1.38, 2.18)
	Not in labor force	10,878,385	2.29 (2.05, 2.55)	2.01 (1.88, 2.16)
Immigrant	Yes	6,293,820		
	No	29,818,810	1.46 (1.38, 1.54)	1.08 (1.05, 1.11)
Indigenous identity	No	34,365,925		
	Yes	1,746,715	0.84 (0.70, 1.01)	1.17 (1.05, 1.30)
Racialized group	No	31,087,995		
	Yes	5,024,640	0.49 (0.44, 0.55)	0.77 (0.73, 0.82)
Occupational	Management	604,425		
	Professional	5,468,800	0.76 (0.48, 1.20)	1.04 (0.75, 1.43)
	Skilled, technical, and supervisory	8,649,120	0.88 (0.57, 1.36)	1.18 (0.86, 1.60)
	Semi-skilled	7,967,015	0.93 (0.60, 1.43)	1.29 (0.95, 1.76)
	Unskilled	4,007,120	1.08 (0.69, 1.70)	1.31 (0.95, 1.81)
	Not applicable	9,416,160	1.97 (1.30, 2.96)	2.23 (1.66, 3.01)
CAN-Marg: residential instability	Q1: lowest	8,423,245		
	Q2	9,716,915	1.08 (1.01, 1.16)	0.98 (0.94, 1.02)
	Q3	7,233,190	1.02 (0.94, 1.09)	0.93 (0.89, 0.97)
	Q4	6,206,875	1.08 (1.00, 1.16)	0.94 (0.90, 0.98)
	Q5: highest	4,532,405	1.11 (1.03, 1.19)	0.91 (0.87, 0.95)
CAN-Marg: ethnic concentration	Q1: lowest	11,721,165		
	Q2	9,231,960	1.13 (1.07, 1.20)	1.08 (1.04, 1.12)
	Q3	6,396,245	0.97 (0.91, 1.04)	1.07 (1.03, 1.12)
	Q4	4,760,510	0.85 (0.79, 0.92)	1.08 (1.03, 1.13)

Covariate	Sub-group	Person-Years (n)[a]	Alzheimer's Disease	Dementia
	Q5: highest	4,002,760	0.70 (0.65, 0.77)	1.06 (1.01, 1.12)
CAN-Marg: material deprivation	Q1: lowest	7,661,965		
	Q2	6,930,790	0.93 (0.87, 1.00)	0.95 (0.91, 0.99)
	Q3	7,182,655	1.01 (0.94, 1.08)	0.93 (0.89, 0.98)
	Q4	6,065,760	0.99 (0.92, 1.07)	0.92 (0.88, 0.96)
	Q5: highest	8,271,465	1.10 (1.03, 1.17)	0.88 (0.85, 0.92)
CAN-Marg: economic dependency	Q1: lowest	6,542,340		
	Q2	5,911,535	1.03 (0.94, 1.13)	0.92 (0.87, 0.97)
	Q3	5,651,030	1.03 (0.94, 1.12)	0.87 (0.83, 0.92)
	Q4	7,235,840	0.94 (0.87, 1.03)	0.86 (0.82, 0.90)
	Q5: highest	10,771,890	1.04 (0.97, 1.13)	0.82 (0.78, 0.86)
Urban form	Active urban core	3,007,995		
	Transit-reliant suburb	2,285,480	0.86 (0.77, 0.95)	0.91 (0.85, 0.97)
	Car-reliant suburb	15,453,930	0.98 (0.91, 1.05)	1.00 (0.96, 1.05)
	Exurban	2,139,055	1.06 (0.94, 1.20)	1.04 (0.96, 1.13)
	Non-CMA/CA	13,226,180	1.00 (0.93, 1.08)	0.92 (0.88, 0.97)
CMA/CA size	Non-CMA/CA	10,441,000		
	Population: 10,000-29,999	6,140,385	1.02 (0.96, 1.09)	1.09 (1.05, 1.14)
	Population: 30,000-99,999	6,805,310	0.90 (0.84, 0.97)	1.17 (1.11, 1.22)
	Population: 100,000-499,999	3,389,785	1.06 (0.99, 1.14)	1.16 (1.11, 1.22)
	Population: 500,000-1,499,999	1,405,345	1.08 (0.99, 1.17)	1.09 (1.03, 1.16)
	Population: >1,500,000	7,930,800	1.04 (0.92, 1.17)	1.16 (1.07, 1.25)
Airshed	East Central	20,986,450		
	Prairie	4,919,345	0.59 (0.54, 0.64)	1.07 (1.02, 1.12)
	West Central	2,057,610	0.67 (0.59, 0.74)	1.13 (1.06, 1.20)
	Southern Atlantic	3,389,670	1.13 (1.05, 1.21)	1.21 (1.15, 1.27)
	Western	20,986,450	0.86 (0.80, 0.92)	1.05 (1.00, 1.09)
	Northern	331,100	1.39 (1.06, 1.83)	0.98 (0.79, 1.22)

[a] Person-years rounded to the nearest whole number

Abbreviations: CMA, census metropolitan area; CA, census area; Q1, quintile 1; Q2, quintile 2; Q3, quintile 3; Q4, quintile 4

Table S4: Source-specific single-pollutant $PM_{2.5}$ effects (per μg/m³) on dementia mortality: comparison of effect direction (hazard ratios, 95% CI) with $PM_{2.5}$ weights from multipollutant models.

Sector	$PM_{2.5}$ Weight	Single-Pollutant HR (95% CI)	Direction Match
**ONRD**	-0.424	0.981 (0.943, 1.021)	Consistent
**OFRD**	0.554	1.059 (1.033, 1.086)	Consistent
**AMR**	-0.348	0.972 (0.959, 0.986)	Consistent
**ORE**	-0.346	0.988 (0.973, 1.002)	Consistent
**EPG**	0.942	1.014 (1.004, 1.024)	Consistent
**MAN**	-0.478	1.009 (0.997, 1.020)	Slight inconsistency (null HR)
**OAG**	0.681	1.053 (1.022, 1.086)	Consistent
**RES**	-0.253	1.113 (1.088, 1.139)	Inconsistent

Note: *Consistency of direction refers to whether the sign of the $PM_{2.5}$ weight from the multipollutant model aligns with the direction of the hazard ratio from the single-pollutant model.*

Abbreviations: AMR, air-marine-rail transportation; CI, confidence interval; EPG, electric power generation; MAN, manufacturing; OAG, oil and gas; OFRD, off-road transportation; ONRD, on-road transportation; ORE, ore and mineral industries; $PM_{2.5}$, particulate matter ≤2.5 micrometers in diameter; RES, residential fuel combustion.

Supplementary References

1. Statistics Canada. Postal Code Conversion File Plus (PCCF+) Version 6D, Reference Guide: August 2015 Postal Codes. Statistics Canada; 2017. Postal Code OM Conversion File Plus (PCCF+)
